# E3-ubiquitin ligase TRIM6 aggravates myocardial ischemia/reperfusion injury via promoting STAT1-dependent cardiomyocyte apoptosis

**DOI:** 10.18632/aging.101995

**Published:** 2019-06-06

**Authors:** Guangwei Zeng, Chen Lian, Pei Yang, Mingming Zheng, He Ren, Haiyan Wang

**Affiliations:** 1 Department of Cardiology, The Second Affiliated Hospital of Air Force Medical University, Shaanxi, China; 2 Jiajiang Oil Storage Warehouse, Xining Joint Service Centre, Xining, China; 3 Department of Health Economic Managment, The Second Affiliated Hospital of Air Force Medical University, Shaanxi, China

**Keywords:** myocardial ischemia, reperfusion injury, E3-ubiquitin ligase TRIM6, STAT1, cardiomyocyte, apoptosis

## Abstract

Cardiomyocyte apoptosis is a major cause of myocardial ischemia/reperfusion (MI/R) injury, in which the activation of the signal transducer and activator of transcription 1 (STAT1) plays an important role. The E3-ubiquitin ligase TRIM6 has been implicated in regulating STAT1 activity, however, whether it is associated with MI/R injury and the underlying mechanism are not determined. In this study, by investigating a mouse MI/R injury model, we show that TRIM6 expression is induced in mouse heart following MI/R injury. Additionally, TRIM6 depletion reduces and its overexpression increases myocardial infarct size, serum creatine phosphokinase (CPK) level and cardiomyocyte apoptosis in mice subjected to MI/R injury, indicating that TRIM6 functions to aggravate MI/R injury. Mechanistically, TRIM6 promotes IKKε-dependent STAT1 activation, and the inhibition of IKKε or STAT1 with the specific inhibitor, CAY10576 or fludarabine, abolishes TRIM6 effects on cardiomyocyte apoptosis and MI/R injury. Similarly, TRIM6 mutant lacking the ability to ubiquitinate IKKε and induce IKKε/STAT1 activation also fails to promote cardiomyocyte apoptosis and MI/R injury. Thus, these results suggest that TRIM6 aggravates MI/R injury through promoting IKKε/STAT1 activation-dependent cardiomyocyte apoptosis, and that TRIM6 might represent a novel therapeutic target for alleviating MI/R injury.

## INTRODUCTION

Myocardial infarction (MI) is one of the leading causes of death and disability worldwide [1]. MI originates from the shortage of myocardial blood supply, which is commonly caused by the intracoronary thrombus-induced occlusion of a coronary artery [2]. To date, the mainstay therapeutic option for reducing myocardial ischemic injury is timely reperfusion. Although reperfusion preserves myocardial viability and function through a reversal of ischemia, itself induces cardiomyocyte death and increases MI size, known as myocardial reperfusion injury, which undermines the therapeutic benefit [3]. Myocardial ischemia/reperfusion (MI/R) injury is a complicated pathological condition and represents a major clinical problem, for which there is still no effective therapy. It is increasingly recognized that the irreversible cardiomyocyte apoptosis plays a critical role in MI/R injury, and therefore, the anti-apoptotic strategy offers a therapeutic opportunity for reducing MI/R injury [4].

The signal transducers and activators of transcription (STAT) family consists of transcriptional factors (STATs) that mediate the intracellular signaling through phosphorylation, dimerization and translocation to the nucleus, which ultimately regulate gene expression [5]. STATs have versatile roles in biological activities, such as homeostasis, development, proliferation and apoptosis, etc, [6]. Previous studies have reported that STAT1 activation induces cardiomyocyte apoptosis following I/R injury by enhancing the expression of pro-apoptotic proteins [7, 8]. In addition, epigallocatechin-3-gallate (EGCG) [9, 10], and myricetin and delphinidin [11] protect cardiomyocytes from MI/R injury-induced apoptosis by inhibiting STAT1 activation. Evidence from in vivo experiments has also shown that STAT1 deficiency in the heart protects against myocardial infarction [12]. These studies suggest that inhibiting STAT1 signaling may be a potential therapy to minimize cardiomyocyte apoptosis following MI/R injury.

The ubiquitin proteasome system (UPS) is a fundamental regulator of protein quality control in almost all mammalian cells, including cardiomyocytes. Among members of UPS, the E3-ubiquitin ligases play a key role in regulating the ubiquitin modification onto target proteins, whereby influencing their protein levels, activity and cellular distribution [13]. The role of E3-ubiquitin ligases involved in the heart health and disease is rapidly expanding [14]. In recent years, several cardiac E3-ubiquitin ligases have been linked to the regulation of cardiomyocyte survival and MI/R injury, such as MDM2 [15], MAFBx [16], MuRF1 [17], and Nrdp1 [18]. One study has shown that TRIM6, a member of the E3-ubiquitin ligase tripartite motif (TRIM) family of proteins, cooperates with the E2-ubiquitin conjugase UbE2K in the synthesis of unanchored K48-linked polyubiquitin chains, which activates IKKε for subsequent STAT1 phosphorylation [19]. Given that STAT1 plays an important role in cardiomyocyte apoptosis following MI/R injury, we hypothesized that TRIM6 may be involved in the regulation of cardiomyocyte apoptosis and MI/R injury.

In the current study, we investigated the role and mechanism of TRIM6 in MI/R injury by utilizing a mouse model developed by the occlusion of left coronary artery (LCA) and subsequent reperfusion. We found that TRIM6 was elevated and functioned to aggravate MI/R injury, which was demonstrated to be mediated by STAT1-promoted cardiomyocyte apoptosis, hence establishing a causal link between an E3-ubiquitin ligase with MI/R injury pathology.

## RESULTS

### TRIM6 expression is induced after MI/R injury

**Figure 1:**
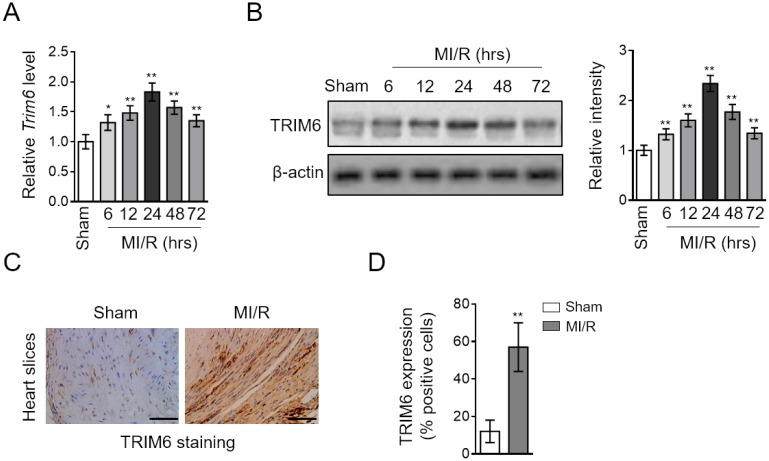
**TRIM6 expression is induced in the heart after MI/R injury.** (**A**–**D**) Mice were subjected to sham surgery or experimental MI/R. Each group contained 7 mice. At different time periods after reperfusion, the hearts were harvested for analyses. (**A**) The mRNA level of *Trim6* in the heart was determined by qRT-PCR analysis. *Actb* was used as an endogenous control. Results relative to sham are shown. (**B**) The protein level of TRIM6 in the heart was determined by Western blotting analysis. β-Actin was used a loading control. The representative images (left) and band intensity relative to sham (right) are shown. (**C**) The expression of TRIM6 in the heart slices at 24 hrs after reperfusion was detected by immunohistochemistry (IHC). Scale bar, 100 μM. (**D**) The quantification of TRIM6 staining shown in (**C**) was shown as percentage of positive staining cardiomyocytes in the heart slices. Data are expressed as mean ± SD (n = 7). **, P < 0.01; *, P < 0.05 vs. sham group.

We initially sought the possible role of TRIM6 involved in MI/R injury by comparing its transcript level in the hearts of mice subjected to sham or experimental MI/R. As a result, qRT-PCR analysis showed that compared with sham group, the transcript level of Trim6 was increased in the heart after MI/R injury, which peaked at 24 hr and then declined thereafter (Figure 1A). Likewise, the protein level of TRIM6 displayed similar tendency of upregulation in the heart of MI/R group, as analyzed by Western blotting assay (Figure 1B). Moreover, the induction of TRIM6 expression was further confirmed by an enhanced staining of TRIM6 in MI/R hearts, as shown by immunohistochemistry analysis (Figure 1C–1D). Altogether, these results indicate that TRIM6 is upregulated at both mRNA and protein levels in the heart following MI/R injury.

### TRIM6 functions to aggravate MI/R injury

**Figure 2:**
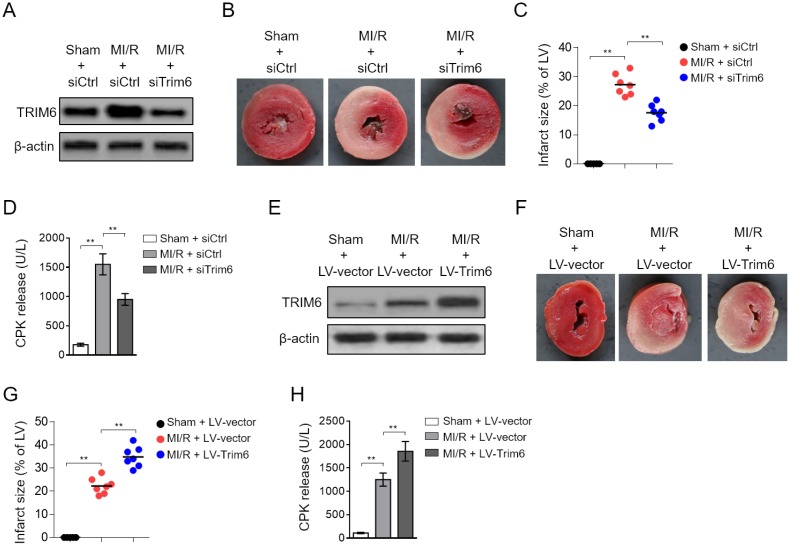
**Cardiac TRIM6 promotes MI/R injury.** (**A**–**D**) The mouse heart was pre-transfected in vivo with control siRNA (siCtrl) or siRNA targeting Trim6 (siTrim6) 48 hrs before surgery. Mice were then subjected to sham surgery or experimental MI/R. Each group contained 7 mice. At 24 hrs after reperfusion, the heart and serum samples were harvested for analyses. (**A**) The protein level of TRIM6 in the heart was determined by Western blotting analysis. β-Actin was used a loading control. (**B**) The mid-myocardial cross sections of hearts were stained with TTC to show infarct size (IS). The representative images are shown. (**C**) The infarct severity in each group shown as in (**B**) is expressed as % of LV (IS/LV). (**D**) The level of serum creatine phosphokinase (CPK) from each group was measured (U/L). (**E**–**H**) The mouse heart was pre-infected in vivo with lentivirus expressing vector control (LV-vector) or Trim6 (LV-Trim6) 48 hrs before surgery. Mice were then subjected to sham surgery or experimental MI/R. Each group contained 7 mice. At 24 hrs after reperfusion, the heart and serum samples were harvested for analyses. (**E**) The protein level of TRIM6 in the heart was determined by Western blotting analysis. β-Actin was used a loading control. (**F**) The mid-myocardial cross sections of heart were stained with TTC. The representative images are shown. (**G**) The quantification of IS in each group (% of LV). (**H**) The level of serum creatine phosphokinase (CPK) from each group was measured (U/L). All data are expressed as mean ± SD (n = 7). **, P < 0.01.

The expression change of TRIM6 occurring after MI/R injury suggests that it may play a functional role in this pathologic condition. To test this, we depleted cardiac TRIM6 by delivering siRNA into the heart, which specifically targets TRIM6. As shown by Western blotting analysis, the TRIM6 in the heart after MI/R was efficiently depleted by siRNA-mediated knockdown (Figure 2A). More importantly, the 2,3,5-triphenyltetrazolium chloride (TTC) staining of the myocardial tissues depicted that the induced infarct size by MI/R injury was substantially diminished when TRIM6 was depleted by siRNA (Figure 2B). Further statistical analysis indicated that the ratio of infarct size (IS) to left ventricular (LV) was drastically decreased in TRIM6-depleted hearts after MI/R injury, as compared with siCtrl group (Figure 2C). Moreover, serving as an index of myocardial injury [20], the increased level of serum creatine phosphokinase (CPK) in mice subjected to MI/R injury was also considerably reduced when cardiac TRIM6 was depleted (Figure 2D), together indicating that TRIM6 knockdown in the heart ameliorates MI/R injury.

Since TRIM6 knockdown ameliorates MI/R injury, which suggests a detrimental role of TRIM6 in this condition. In order to directly demonstrate it, we overexpressed TRIM6 in the heart by lentiviral infection. Western blotting analysis showed that compared with vector control (LV-vector), lentivirus infection-mediated TRIM6 overexpression (LV-Trim6) resulted in stark increase in TRIM6 level in the heart (Figure 2E). The staining of myocardial tissues showed an exacerbated infarct size in TRIM6-overexpression heart after MI/R injury (Figure 2F). Additionally, the ratio of IS to LV was increased in LV-Trim6 group after MI/R injury, as compared with LV-vector group (Figure 2G). Consistent with the exacerbated MI/R injury, the serum CPK level was further increased in MI/R mice by TRIM6 overexpression (Figure 2H). Thus, these data prove that TRIM6 overexpression in the heart promotes MI/R injury, together with the result that TRIM6 knockdown ameliorates MI/R injury, pointing to an adverse role of TRIM6 involved in MI/R injury.

### TRIM6 promotes myocardial apoptosis after MI/R injury

**Figure 3:**
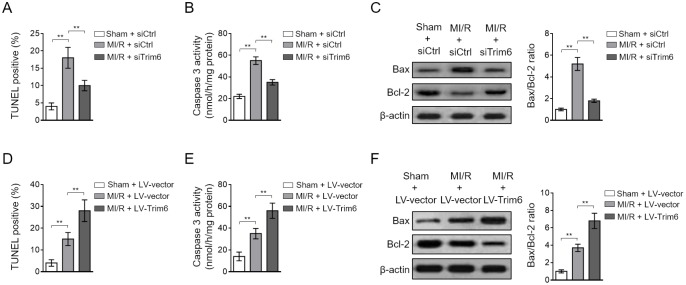
**TRIM6 promotes myocardial apoptosis after MI/R injury.** (**A**–**C**) The mouse heart was pre-transfected in vivo with control siRNA (siCtrl) or siRNA targeting *Trim6* (siTrim6) 48 hrs before surgery. Mice were then subjected to sham surgery or experimental MI/R. Each group contained 7 mice. At 24 hrs after reperfusion, the hearts were harvested for analyses. (**A**) The myocardial apoptosis in the heart sections was identified by TUNEL staining. The quantitative analysis of TUNEL-positive cardiomyocytes is shown (%). (**B**) The caspase-3 activity of the heart was measured and expressed as nmol per hr per mg protein. (**C**) The protein expression of Bax and Bcl-2 was determined by Western blotting analysis. β-Actin was used a loading control. The representative images (left) and the Bax/Bcl-2 ratio relative to sham (right) are shown. (**D**–**F**) The mouse heart was pre-infected in vivo with lentivirus expressing vector control (LV-vector) or Trim6 (LV-Trim6) 48 hrs before surgery. Mice were then subjected to sham surgery or experimental MI/R. Each group contained 7 mice. At 24 hrs after reperfusion, the hearts were harvested for analyses. The myocardial apoptosis (**D**), caspase-3 activity (**E**), and Bax/Bcl-2 ratio (**F**) in the heart were analyzed as in (**A**–**C**). All data are expressed as mean ± SD. **, P < 0.01.

The induction of myocardial apoptosis is fundamental for MI/R injury [21]. We found that siRNA-mediated TRIM6 knockdown minimized myocardial apoptosis induced by MI/R injury, as evidenced by the decreased number of TUNEL positive cardiomyocytes (Figure 3A), reduced caspase 3 activity (Figure 3B) and expression of cleaved caspase-3 (Supplementary Figure 2A), as well as declined Bax/Bcl-2 ratio (Figure 3C). In contrast, TRIM6 overexpression in the heart of MI/R mice increased the number of TUNEL positive cardiomyocytes (Figure 3D) and elevated the caspase 3 activity (Figure 3E) and expression of cleaved caspase-3 (Supplementary Figure 2B), and Bax/Bcl-2 ratio (Figure 3F). Taken together, these findings illustrate that TRIM6 promotes myocardial apoptosis after MI/R injury, which is in accordance with its detrimental role in exacerbating MI/R injury shown in Figure 2.

### TRIM6 promotes myocardial apoptosis via inducing IKKε-dependent STAT1 activation after MI/R injury

**Figure 4:**
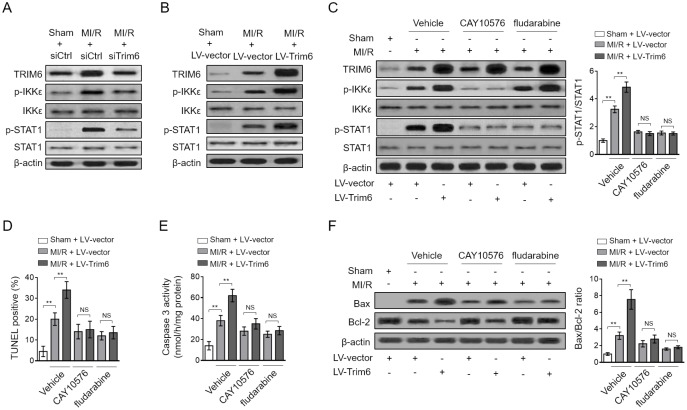
**TRIM6 promotes myocardial apoptosis through inducing IKKε-mediated STAT-1 activation after MI/R injury.** (**A**) The mouse heart was pre-transfected in vivo with control siRNA (siCtrl) or siRNA targeting Trim6 (siTrim6) 48 hrs before surgery. Mice were then subjected to sham surgery or experimental MI/R. Each group contained 7 mice. At 24 hrs after reperfusion, the hearts were harvested for analyses. The protein expression of TRIM6, p-IKKε, IKKε, p-STAT1 and STAT1 in the heart was determined by Western blotting analysis. β-Actin was used a loading control. (**B**) The mouse heart was pre-infected in vivo with lentivirus expressing vector control (LV-vector) or Trim6 (LV-Trim6) 48 hrs before surgery. Mice were then subjected to sham surgery or experimental MI/R. Each group contained 7 mice. At 24 hrs after reperfusion, the hearts were harvested for analyses. The protein expression of TRIM6, p-IKKε, IKKε, p-STAT1 and STAT1 in the heart was determined by Western blotting analysis. β-Actin was used a loading control. (**C**–**F**) The mouse heart was pre-infected in vivo with lentivirus expressing vector control (LV-vector) or Trim6 (LV-Trim6) in the presence or absence of CAY10576 or fludarabine 48 hrs before surgery. Mice were then subjected to sham surgery or experimental MI/R. Each group contained 7 mice. At 24 hrs after reperfusion, the hearts were harvested for analyses. (**C**) The protein expression of TRIM6, p-IKKε, IKKε, p-STAT1 and STAT1 in the heart was determined by Western blotting analysis. β-Actin was used a loading control. Shown here are representative images (left) and the p-STAT1/STAT1 ratio relative to sham (right). (**D**) The myocardial apoptosis in the heart sections was identified by TUNEL staining. The quantitative analysis of TUNEL-positive cardiomyocytes is shown (%). (**E**) The caspase-3 activity of the heart was measured and expressed as nmol per hr per mg protein. (**F**) The protein expression of Bax and Bcl-2 was determined by Western blotting analysis. β-Actin was used a loading control. The representative images (left) and the Bax/Bcl-2 ratio relative to sham (right) are shown. All data are expressed as mean ± SD (n = 7). **, P < 0.01; NS, not significant.

Myocardial apoptosis is orchestrated by diverse signal transduction pathways, in which STAT1 activation plays a critical role [22]. Besides, TRIM6 has been implicated in the regulation of STAT1 activation in an IKKε-dependent manner [19]. To elucidate the molecular mechanisms by which TRIM6 promotes myocardial apoptosis, we next examined whether IKKε/STAT1 axis participates in this pathological condition. Coinciding with a previous study [10], we noticed that the phosphorylated levels of IKKε and STAT1 were increased after MI/R injury, indicating that the IKKε/STAT1 pathway is activated in response to MI/R injury (Figure 4A). Moreover, intriguingly, TRIM6 depletion largely suppressed their activation (Figure 4A), and oppositely, TRIM6 overexpression increased the phosphorylation levels of IKKε and STAT1 (Figure 4B). These lines of evidence indicate that the activation of IKKε/STAT1 pathway after MI/R injury is positively regulated by TRIM6.

To gain insights into the functional role of IKKε/STAT1 pathway in TRIM6-promoted myocardial apoptosis, the specific inhibitor of IKKε (CAY10576) and STAT1 (fludarabine) were used to inhibit this signaling pathway [23, 24]. The results showed that the promoted activation of IKKε/STAT1 pathway by TRIM6 overexpression in the heart of MI/R mice was efficiently inhibited by both CAY10576 and fludarabine (Figure 4C). Notably, keeping in line with the inhibited IKKε/STAT1 activation, the promoted myocardial apoptosis by TRIM6 overexpression was totally abolished, as shown by the results of TUNEL assay (Figure 4D), caspase 3 activity (Figure 4E), and Bax/Bcl-2 ratio (Figure 4F). Collectively, these observations indicate that TRIM6 promotes myocardial apoptosis via inducing IKKε-dependent STAT1 activation after MI/R injury.

### TRIM6 overexpression-promoted MI/R injury is abrogated by inhibiting IKKε/STAT1 axis

**Figure 5:**
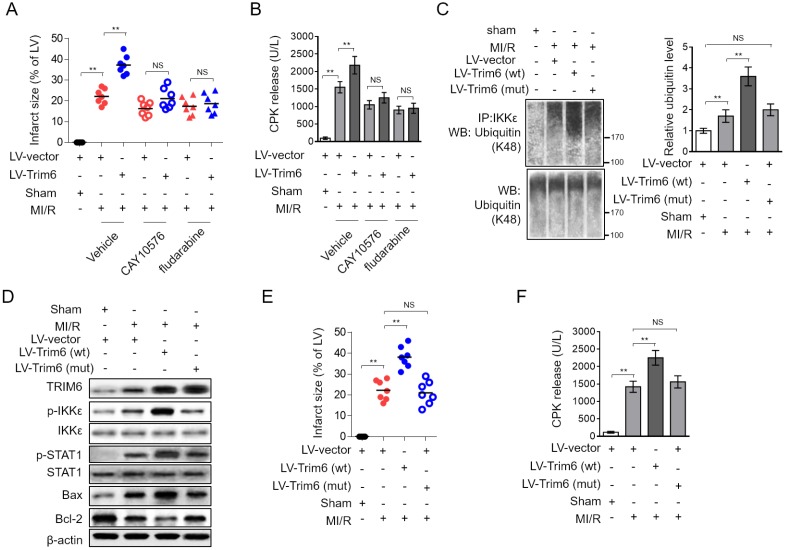
**Inhibition of IKKε/STAT-1 axis abrogates the promoted MI/R injury by TRIM6 overexpression**. (**A**–**B**) The mouse heart was pre-infected in vivo with lentivirus expressing vector control (LV-vector) or Trim6 (LV-Trim6) in the presence or absence of CAY10576 or fludarabine 48 hrs before surgery. Mice were then subjected to sham surgery or experimental MI/R. Each group contained 7 mice. At 24 hrs after reperfusion, the hearts were harvested for analyses. (**A**) The mid-myocardial cross sections of heart were stained with TTC, and the quantification of infarct size in each group (% of LV) is shown. (**B**) The level of serum creatine phosphokinase (CPK) from each group was measured. (**C**–**F**) The mouse heart was pre-infected in vivo with lentivirus expressing vector control (LV-vector), wild-type Trim6 (LV-Trim6-wt) or C15A RING mutant Trim6 (LV-Trim6-mut) 48 hrs before surgery. Mice were then subjected to sham surgery or experimental MI/R. Each group contained 7 mice. At 24 hrs after reperfusion, the hearts were harvested for analyses. (**C**) The lysates of heart tissues were co-immunoprecipitated (co-IP) by IKKε antibody. The expression of ubiquitin (K48) in the co-IP products and input samples was measured by Western blotting analysis. (**D**) The protein expression of TRIM6, p-IKKε, IKKε, p-STAT1, STAT1, Bax and Bcl-2 in the heart was determined by Western blotting analysis. β-Actin was used a loading control. (**E**) The mid-myocardial cross sections of heart were stained with TTC, and the quantification of infarct size in each group (% of LV) is shown. (**F**) The level of serum creatine phosphokinase (CPK) from each group was measured. The All data are expressed as mean ± SD (n = 7). **, P < 0.01; NS, not significant.

**Figure 6:**
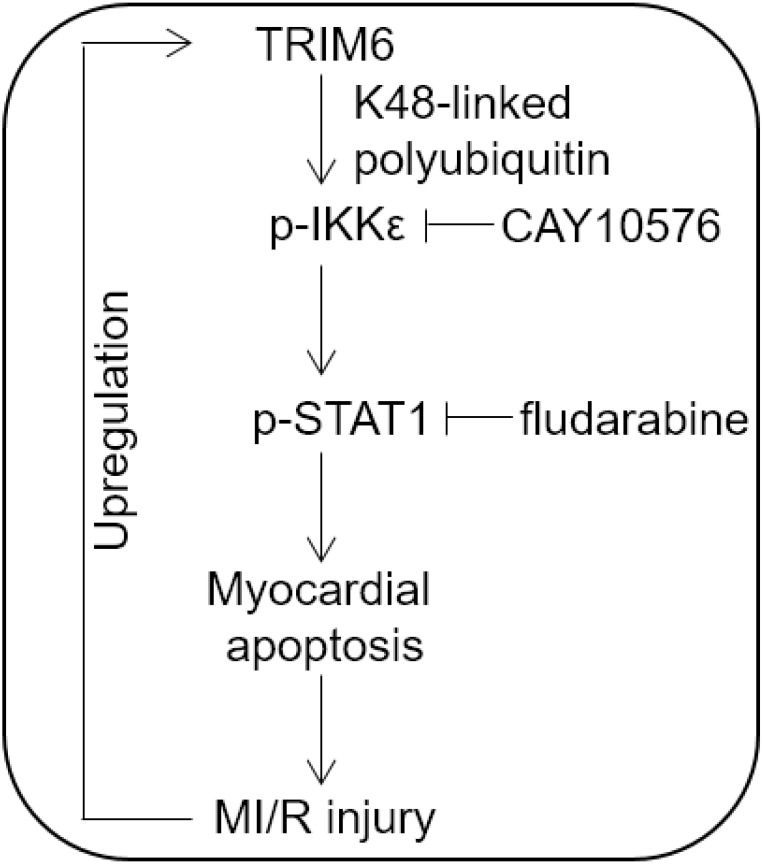
**Proposed model of this study.** Brief schematic diagram of the interplay between TRIM6 and MI/R injury. TRIM6 induces the activation of IKKε through K48-linked polyubiquitination and further activates p-STAT1, thus promoting myocardial apoptosis and MI/R injury, which in turn leads to the upregulation of TRIM6 via an unknown mechanism.

To understand the contribution of IKKε-dependent STAT1 activation to TRIM6-promoted MI/R injury, we assessed the infarct size and serum CPK level when IKKε/STAT1 axis was blocked by CAY10576 or fludarabine. We found that the treatment of both CAY10576 and fludarabine reversed TRIM6-aggravated myocardial infarct size (Figure 5A) and serum CPK level (Figure 5B), therefore illustrating that TRIM6 promotes MI/R injury by inducing IKKε/STAT1 activation-mediated myocardial apoptosis. It has been previously demonstrated that TRIM6 induces IKKε/STAT1 activation through catalyzing the modification of unanchored K48-linked polyubiquitin chains to IKKε, which requires its E3 ligase activity [19]. To gain further mechanistical insights into the role of TRIM6-mediated IKKε/STAT1 activation in MI/R injury, a C15A RING mutant TRIM6 (LV-Trim6-mut) was constructed, which lacks the E3 catalytic activity to catalyze IKKε polyubiquitin [19]. Co-immunoprecipitation (Co-IP) assay demonstrated that the K48-linked polyubiquitin bound to IKKε was increased in the heart following MI/R injury, which was further enhanced by the overexpression of wild-type Trim6 (LV-Trim6-wt) but not LV-Trim6-mut (Figure 5C), indicating that TRIM6 catalyzes IKKε ubiquitination following MI/R injury. Notably, consistent with the notion that TRIM6 activates IKKε/STAT1 through IKKε ubiquitination, we found that compared with LV-Trim6-wt overexpression, LV-Trim6-mut overexpression failed to enhance the activation of IKKε/STAT1 in the heart after MI/R injury (Figure 5D). Accordingly, Western blotting analysis of Bax and Bcl-2 showed that the promoted myocardial apoptosis after MI/R injury also vanished when overexpressed with LV-Trim6-mut (Figure 5D). Furthermore, in concert with this result, LV-Trim6-mut overexpression was unable to aggravate MI/R injury, as evidenced by the results of infarct size (Figure 5E) and CPK release (Figure 5F). Thus, these lines of evidence further consolidate the concept that TRIM6 promotes myocardial apoptosis and exacerbates MI/R injury through inducing the activation of IKKε/STAT1 axis, which requires its E3 ligase activity to catalyze K48-linked polyubiquitin of IKKε (Figure 6).

## DISCUSSION

MI/R injury is referred to the consequence of the damage and subsequent cell death induced by the procedure of reperfusion therapy. It has been well established that the myocardial cell death occurs via two independent models of death following MI/R injury, i.e., apoptosis and necrosis [25, 26]. Particularly, two central pathways, the receptor-mediated (extrinsic) and the mitochondrial (intrinsic) which lead to the execution of myocardial apoptosis, hold the promise of serving as the targets for reducing infarct size and preserving myocardial function following MI/R injury [27]. As such, advancing a comprehensive understanding of the molecular mechanisms underlying the myocardial apoptosis becomes an important task. It has long been recognized that the signal transduction pathways through which MI/R leads to myocardial apoptosis involve the activation of JNK and JAK/STAT pathways, generation of ceramide and the inhibition of PKC pathway, and accordingly, the biochemical events are triggered, including mitochondrial impairment, cytochrome c release, caspase cascade activation and cytoplasmic acidification [22, 28]. However, the regulatory paradigm remains uncompleted and new regulators are increasingly uncovered. Among these, the cardiac ubiquitin ligases attract much attention [14, 29, 30]. To date, the role of ubiquitin ligases like Atrogin1, MuRF1, CHIP, Parkin, Nrdp1 and MDM2 in regulating the myocardial apoptosis and MI/R injury via signaling pathways, such as JNK, AKT, STAT, MAPK and NF-κB, etc, and their potential as therapeutic targets have recently been reported [14]. Here, in the current study, we show that an E3-ubiquitin ligase TRIM6 induces myocardial apoptosis and aggravates MI/R injury by promoting IKKε-dependent STAT1 activation, thus offering a novel role and mechanism by which TRIM6 regulates myocardial apoptosis and MI/R injury (Figure 6). These results also support IKKε/STAT1 axis as another important signal transduction in positively regulating the myocardial apoptosis following MI/R injury.

We initially found that TRIM6 was upregulated in the heart following MI/R injury. Of note, its mRNA level and protein level were both upregulated, suggesting a transcriptional induction in the heart tissue was launched in response to MI/R injury. However, at present, the upstream pathways and transcriptional factors that are responsible for regulating TRIM6 transcription are less known. It’s reported that many TRIM family members are inducible by interferons in the context of innate immune responses [31], this might be of certain relevance to the upregulation of TRIM6 due to the inflammatory conditions of the heart following MI/R injury [32]. Generally, MI/R is a pathological condition characterized by an initial shortage of blood supply to the myocardium, whereby causing tissue hypoxia, and the following restoration of perfusion and concomitant reoxygenation frequently result in tissue injury and a profound inflammatory response [33]. We suspect that the transcriptional activation of TRIM6 is probably stimulated by these pathological conditions, such as local hypoxia and pro-inflammatory factors, and that the observed decline of TRIM6 expression at 48 hrs after reperfusion may be explained by the alleviated stimulation. In vitro evidence from hypoxia/reoxygenation assay using cultured cardiomyocytes in the presence or absence of pro-inflammatory factors would be helpful to providea hint.

We noticed that both knockdown (Supplementary Figure 1A–1B) and overexpression (Supplementary Figure 1C–1D) of TRIM6 alone did not obviously affect myocardial apoptosis and infarct size in sham-operated mice, suggesting that TRIM6 may be functionally dispensable in the heart under normal physiological conditions. Whereas, we further demonstrated that TRIM6 promotes myocardial apoptosis and increases infarct size via IKKε-mediated STAT1 activation in mice subjected to MI/R injury. This is confirmative, since the blockade of this signaling pathway by either IKKε inhibitor CAY10576 or STAT1 inhibitor fludarabine is sufficient to reverse the TRIM6-promoted myocardial apoptosis and -exaggerated MI/R injury. It has been finely demonstrated in a previous study that TRIM6 activates IKKε for subsequent STAT1 phosphorylation by cooperating with E2-ubiqiutin conjugase UbE2K to synthesize unanchored K48-linked polyubiquitin chains [19]. This mechanism could also be applied to explain our observations that TRIM6 promotes IKKε ubiquitination and activates the pro-apoptotic IKKε/STAT1 pathway in the heart following MI/R injury. It has also been suggested that the phosphorylation site on STAT1 (S708) targeted by IKKε is different from IKKε-independent STAT1 phosphorylation (Y701) mediated by JAK1 and TYK2 [19]. Therefore, the myocardial apoptosis controlled by TRIM6/IKKε/STAT1 axis may be independent from that regulated by JAK/STAT1 pathway [34]. However, whether there exists an interplay between these two pathways is unknown at present. Interestingly, based on the results that the treatment of CAY10576 and fludarabine gives rise to comparable recovery of myocardial apoptosis and infarct size exaggerated by TRIM6, we speculate that under this experimental condition, there might exist a mechanism leading to a convergence of the output of STAT1 signal transduced from IKKε and other upstream factors, e.g., JAK [35]. Elucidating this puzzle could provide insights into the regulaiton of STAT1 following MI/R injury. Another issue is that except for IKKε, other molecules that participate in TRIM6-modulated STAT1 cannot be easily ruled out. For example, β-arrestin 2 inhibits STAT1 activation in macrophages [36], and improves post-myocardial infarction heart failure [37], which imply that it may exert an opposite role in MI/R injury as relative to TRIM6. Further efforts are required to test whether β-arrestin 2 is involved in STAT1 regulation and affects MI/R injury.

Clinical observations indicate that myocardial apoptosis contributes significantly to MI/R injury in humans [28, 38]. In a recent phase 2 clinical trial, the inhibition of apoptosis with cyclosporine has proven efficacy in reducing MI/R injury [39]. In our study, we show that IKKε/STAT1 pathway inhibitors, including CAY10576 and fludarabine, reduce cardiomyocyte apoptosis and MI/R injury in a mouse model, thus offering them as potential candidates in clinical trial for MI/R injury.

Furthermore, it should be noted that there are some limitations in the current study which remain to be addressed by future studies. First, although the mouse MI/R injury model developed by LCA occlusion and following reperfusion displays numerous pivotal pathophysiologic characteristics of clinically relevant MI/R, such as myocardial apoptosis, iron accumulation and immune activation, it does not always faithfully recapitulate the whole disease processes in humans [40]. Investigations using other animal models and human samples are required to consolidate the regulation, functional role and mechanisms of TRIM6 in MI/R injury, which would provide more evidence associating TRIM6 with the pathophysiology of MI/R injury. Second, age- and gender-associated factors influence the susceptibility to MI/R injury [41], however, we merely utilized young male mice, therefore whether TRIM6 exerts similar effects on MI/R injury in aged or female mice is uncertain. Third, apart from its expression, whether the enzymatic activity of TRIM6 is also altered following MI/R injury is unclear. More in-depth studies are warranted to address these issues.

In summary, based on the results we obtained from the MI/R animal model, we propose here that inhibiting TRIM6 expression and/or activity or interfering IKKε-dependent STAT1 activation may present a potential therapeutic option in reducing the size of myocardial infarct and alleviating MI/R injury.

## MATERIALS AND METHODS

### Antibodies and reagents

The antibodies and reagents were obtained from the following sources: TRIM6 (Sigma, SAB1306751), β-Actin (Santa Cruz, AC-15), PARP (Cell Signaling, 9542), Bax (Cell Signaling, 2772), Bcl-2 (Santa Cruz, sc-7382), p-IKKε (Ser172) (Cell Signaling, 8766), IKKε (Cell Signaling, 2690), p-STAT-1 (Tyr701) (Cell Signaling, 9167), STAT1 (Cell Signaling, 9172), Ubiquitin (linkage-specific K48) (abcam, EP8589), goat anti-rabbit IgG H&L (HRP) (abcam, ab6721), goat anti-mouse IgG H&L (HRP) (abcam, ab6789), peroxidase-conjugated goat anti-rabbit antibody (Millipore, AP132P), CAY10576 (Santa Cruz, sc-223870A), fludarabine (Biotechne, 3495), 2,3,5-triphenyltetrazolium chloride (Sigma, T8877).

### Animals and MI/R model

The animal study design and experiments were approved by the Institutional Animal Care and Use Committees of The Second Affiliated Hospital of Air Force Medical University. Twelve-week-old C57BL/6 male mice were used in this study and maintained under specific pathogen-free conditions. Before experimental surgery, mice were randomly divided into different groups underwent sham operation or MI/R injury. Each group contained 7 mice. The animal model of MI/R was established as previously described [4]. In brief, to prevent clot formation, mice were pre-injected with 200 units/kg sodium heparin 1 hr before anesthetization with sodium pentobarbital (50 mg/kg) and ketamine (50 mg/kg). A ventilator (HugoSachs, Model 845) was utilized to ventilate mice with 100% oxygen (0.5 liter/min). The ribcage was cut open with a midline incision along the sternum, followed by thoracotomy at the left of midline performed using an electrocautery. For visualizing the left coronary artery (LCA), a vertical opening was made by cauterizing the second and third ribs. Under the sight of a stereomicroscope (Olympus, SZ61), the LCA was ligated using a 7-0 silk suture to render the left ventricle be ischemic for 30 min. After that, 7-0 silk suture was removed to reperfuse the LCA for further 24 hrs. Mice in control group received sham surgery without procedures of coronary artery ligation.

### qRT-PCR analysis

The heart tissues were harvested from the ischemic border area 24 hrs after reperfusion and immediately frozen in liquid nitrogen. Each group contained 7 mice. The total RNA of mouse heart tissues were isolated using RNeasy Mini Kit (Qiagen, 74106), and 2 μg total RNA were reverted into complimentary cDNA by RevertAid First Strand cDNA Synthesis Kit (ThermoFisher Scientific, K1621) following the manufacturer’s instructions. The transcript level of target genes was quantified with QuantiTect SYBR Green PCR kit (Qiagen, 204143) and CFX96 Real Time PCR instrument (Bio-Rad). The level of mouse *Actb* gene was used as an endogenous control. The primer pairs are listed as follows: Trim6 forward 5′-TCCAGGTATCAGCCACAGTG-3′, reverse 5′-TAGGGACAAAGGGCAGAAGG-3′; Actb forward 5′-CACCATTGGCAATGAGCGGTTC-3′, reverse 5′-AGGTCTTTGCGGATGTCCACGT-3′.

### Western blotting

The heart tissues were harvested from the ischemic border area 24 hrs after reperfusion and immediately frozen in liquid nitrogen. Each group contained 7 mice. The total protein samples of the heart tissues were extracted using Cell Lysis and Protein Extraction Kit (Solarbio, BC3640) according to manufacturer’s instructions. The lysis buffer was added with protease inhibitors cocktail (1:100, Roche). The extraction was performed on ice for 20 min. The cell lysates were centrifuged at 13000 rpm for 10 min at 4 °C to obtain supernatants. The protein concentration was quantified using Bradford Protein Assay Kit (Beyotime, P0006). After quantification, 5 × SDS loading buffer was added into supernatants and proteins were denatured at 100 °C for 5 min. The procedures of Western blotting were conducted as described previously [43]. Briefly, equal amounts of total protein samples were loaded and separated by SDS-PAGE. Then, proteins in the gel were transferred onto the nitrocellulose (NC) membranes (Millipore). After transfer, the NC membranes were blocked with 5% skim milk (BD) for 1 hr at room temperature (RT) and then sequentially incubated overnight with appropriate amount of primary and secondary antibodies at 4 °C. After washed with TBST, NC membranes were incubated with the reagents of ECL Detection Kit (ThermoFisher Scientific, 32106). The protein bands on NC membranes were detected by C600 imaging system (Azure Biosystems). The band intensity was analyzed by ImageJ software.

### Immunohistochemistry

Immunohistochemistry on heart tissues was performed as previously documented [44]. In brief, the heart tissues were harvested from the ischemic border area 24 hrs after reperfusion and 5 μm histological sections were prepared. Each group contained 7 mice, and 4 sequentially cut sections were obtained from each mouse. The heart sections were incubated overnight with the antibody against TRIM6 (1:200 dilution) or IgG isotype control antibody (1:200 dilution) at 4 °C. After mild wash, the heart sections were incubated with peroxidase-conjugated goat anti-rabbit antibody (1:1000). The peroxidase activity was assessed through 2 min reaction with diaminobenzidine (DAB). The heart sections were further counterstained with hematoxylin and observed under a light microscope (Olympus BX51). For quantification analysis, the number of cardiomyocytes positively stained with TRIM6 was counted and expressed as a percentage of the total number of cardiomyocytes in 5 random fields from each section. The mean value of each mouse was calculated.

### Infarct size and serum CPK measurement

The 2,3,5-triphenyltetrazolium chloride (TTC) staining was applied to determine the size of myocardial infarct area [45]. The hearts were removed and washed with PBS and cut it into 2-mm four transverse slices. The slices were stained with 1 ml 1.5% TTC for 15 min at 37 °C to reveal the infarct area. The stained hearts were imaged under a light microscope (Olympus BX51). The size of the left ventricular (LV) area and infarct area (IA) was determined using Image J software. The myocardial infarct size was expressed as % of the LV. Additionally, after MI/R surgery, mice were sacrificed and the blood samples were harvested. The serum was isolated and stored at -80 °C. As an index of myocyte injury, the level of serum creatine phosphokinase in serum samples was determined using Creatine Kinase (CK) kit (Catachem, V184-12) according to the manufactural instructions.

### TUNEL staining and caspase 3 activity assay

The myocardial apoptosis on the heart tissue sections was qualitatively analyzed by TUNEL (terminal deoxynucleotidyl transferase dUTP nick-end labeling) staining assay using DeadEnd Fluorometric TUNEL System (Promega, G3250) as previously reported [46]. Eventually, 5 random microscopic fields from 3 different sections for each mouse were analyzed. The caspase-3 activity was measured using Ac-DEVD-AFC Caspase-3 Fluorogenic Substrate kit (BD Biosciences, 556574) according to manufacturers’ instructions. Briefly, the heart tissues were lysed on ice and supernatants were collected. The protein concentration was quantified. The assay was performed in reaction buffer containing 10 mM DTT and 50 μg total proteins. The fluorescence emission of the AFC was detected by Spectra Max-Plus Microplate Spectrophotometer (Molecular Devices, 400 nm excitation, 505 nm emission). The caspase-3 activity was indicated as nmol AFC/h/mg protein.

### In vivo injection of siRNA, lentivirus and chemical inhibitors

siRNA targeting mouse Trim6 (siTrim6) and non-specific control siRNA (siCtrl) were purchased from Invitrogen. siRNAs were delivered into the local heart tissues by in vivo jet PEI Delivery Reagent (Polyplus, 201-10G) via 3 separate intra-myocardial injections using a 32.5-gauge needle as described previously [47]. For gene overexpression in the heart, the lentiviral particles expressing mouse wild-type Trim6 (LV-Trim6-wt), mouse C15A RING mutant Trim6 (LV-Trim6-mut) or empty vector control (LV-vector) were injected into the mouse left ventricle (30 μl per mouse). To inhibit IKKε and STAT1 in the heart, CAY10576 (200 mg/kg) and fludarabine (100 mg/kg) were injected simultaneously with the lentiviral particles into the mouse left ventricle 48 hrs before MI/R injury. The efficacy of knockdown and overexpression and the inhibitory effect of chemical inhibitors were confirmed by Western blotting analysis.

### Statistics

All values are presented as mean ± SD. Two sets of data were compared using non-parametric Mann-Whitney U test with the aid of GraphPad Prism 6 statistic software. P values less than 0.05 were considered to be statistically significant.

## Supplementary Material

Supplementary Figures
