# Supplementary material for: E3-ubiquitin ligase TRIM6 aggravates myocardial ischemia/reperfusion injury via promoting STAT1-dependent cardiomyocyte apoptosis
Source: Aging (Albany NY). 2019 Jun 6;11(11):3536–50. doi: 10.18632/aging.101995 (PMC6594808; doi:10.18632/aging.101995)
Supplement: Supplementary Figures [file aging-11-101995-s001.pdf]

SUPPLEMENTARY MATERIALS

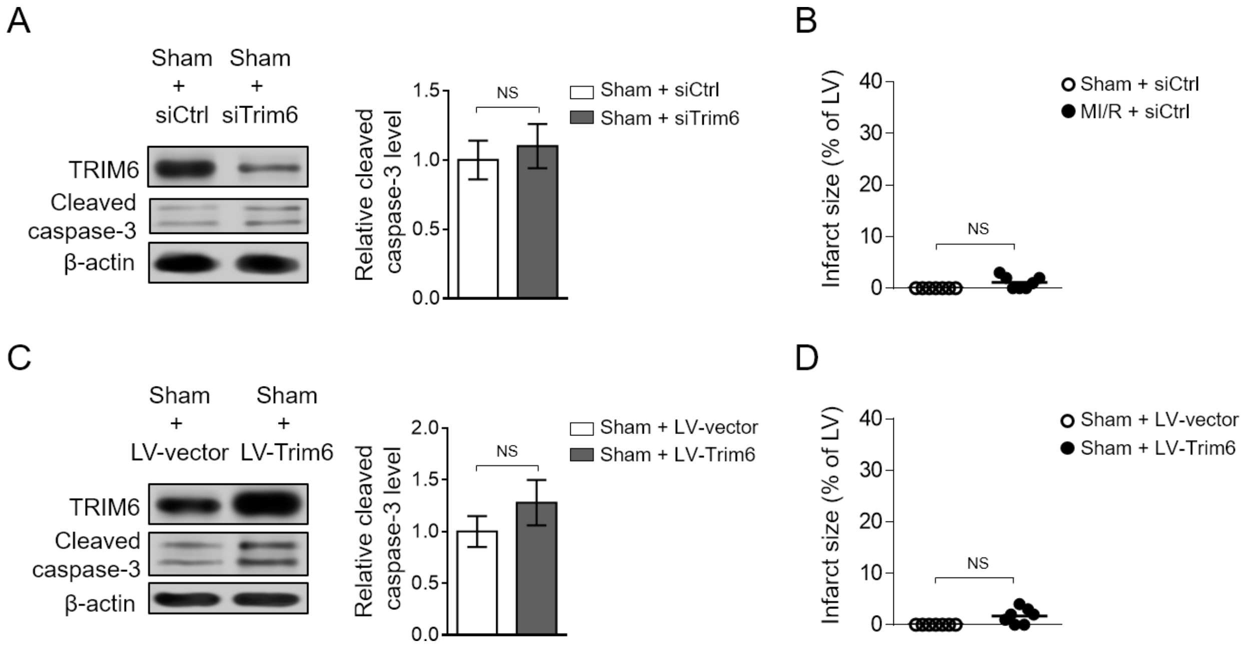

Supplementary Figure 1. TRIM6 manipulation alone does not have obvious effects on the hearts of sham mice. (**A**–**B**) The mouse heart was pre-transfected in vivo with control siRNA (siCtrl) or siRNA targeting Trim6 (siTrim6) for 48 hrs. Mice were then subjected to sham surgery. Each group contained 7 mice. At 24 hrs after reperfusion, the heart was harvested for analyses. (**A**) The protein levels of TRIM6 and cleaved caspase-3 in the heart were determined by Western blotting analysis. β-Actin was used a loading control. (**B**) The infarct size relative to the left ventricular of mid-myocardial cross sections of each group is shown. (**C**–**D**) The mouse heart was pre-infected in vivo with lentivirus expressing vector control (LV-vector) or Trim6 (LV-Trim6) for 48 hrs. Mice were then subjected to sham surgery. Each group contained 7 mice. At 24 hrs after reperfusion, the heart was harvested for analyzing protein expression (**C**) and infarct size (**D**) as described in (**A**–**B**). All data are expressed as mean ± SD (n = 7). NS, not significant.

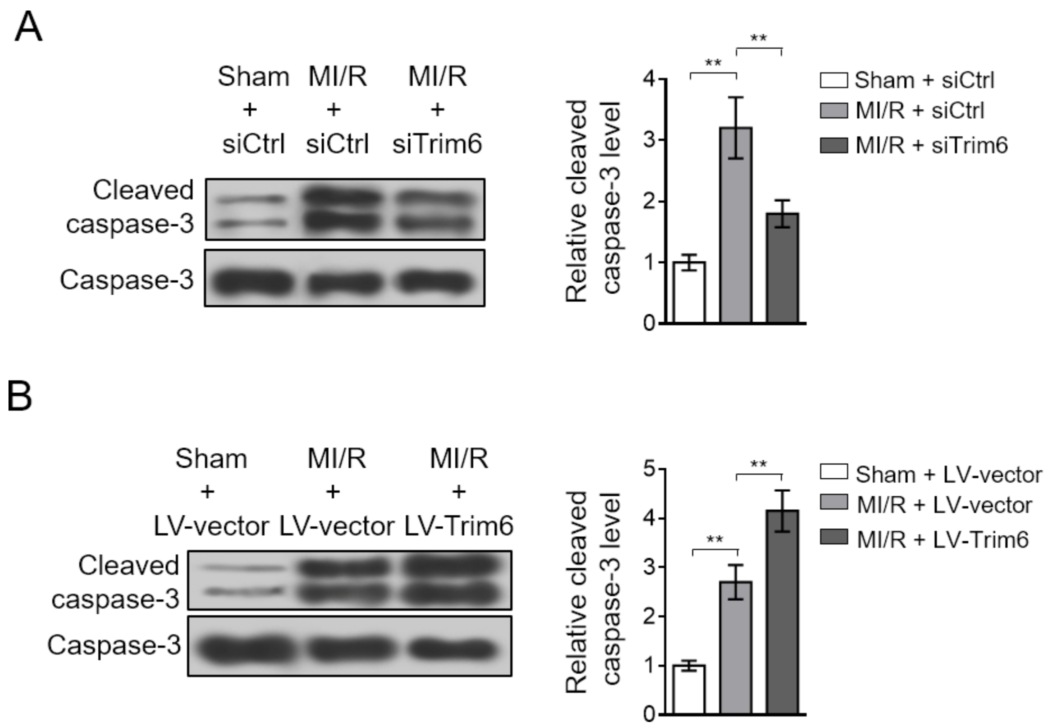

Supplementary Figure 2. TRIM6 increases expression of cleaved caspase-3 after MI/R injury. (**A**) The mouse heart was pre-transfected in vivo with control siRNA (siCtrl) or siRNA targeting Trim6 (siTrim6) for 48 hrs. Mice were then subjected to sham surgery or experimental MI/R. Each group contained 7 mice. At 24 hrs after reperfusion, the hearts were harvested for analyses. The protein expression of cleaved caspase-3 and caspase-3 was determined by Western blotting analysis. The representative images (left) and cleaved caspase-3/caspase-3 ratio relative to sham (right) are shown. (**B**) The mouse heart was pre-infected in vivo with lentivirus expressing vector control (LV-vector) or Trim6 (LV-Trim6) for 48 hrs. Mice were then subjected to sham surgery or experimental MI/R. Each group contained 7 mice. At 24 hrs after reperfusion, the hearts were harvested for analyses. The protein expression was analyzed as in (**A**). All data are expressed as mean ± SD. **, $P < 0.01$.
